# Evidence of Inflammatory Network Disruption in Chronic Venous Disease: An Analysis of Circulating Cytokines and Chemokines

**DOI:** 10.3390/biomedicines13010150

**Published:** 2025-01-09

**Authors:** Oscar Fraile-Martinez, Cielo García-Montero, Ana María Gomez-Lahoz, Felipe Sainz, Julia Bujan, Silvestra Barrena-Blázquez, Laura López-González, Raul Díaz-Pedrero, Melchor Álvarez-Mon, Natalio García-Honduvilla, Miguel A. Saez, Jorge Monserrat, Miguel A. Ortega

**Affiliations:** 1 Department of Medicine and Medical Specialities (CIBEREHD), Faculty of Medicine and Health Sciences, University of Alcalá, 28801 Alcala de Henares, Spain; oscarfra.7@gmail.com (O.F.-M.); cielo.gmontero@gmail.com (C.G.-M.); alahoz1199@gmail.com (A.M.G.-L.); sainz.felipe@gmail.com (F.S.); mjulia.bujan@uah.es (J.B.); mademons@gmail.com (M.Á.-M.); natalio.garcia@uah.es (N.G.-H.); msaega1@oc.mde.es (M.A.S.); jorge.monserrat@uah.es (J.M.); 2 Ramón y Cajal Institute of Sanitary Research (IRYCIS), 28034 Madrid, Spain; silvebarrena@gmail.com (S.B.-B.); laura.lgonzalez@uah.es (L.L.-G.); raul.diazp@uah.es (R.D.-P.); 3 Angiology and Vascular Surgery Service, Central University Hospital of Defence—UAH, 28047 Madrid, Spain; 4 Department of Nursing and Physiotherapy, Faculty of Medicine and Health Sciences, University of Alcalá, 28801 Alcala de Henares, Spain; 5 Department of Surgery, Medical and Social Sciences, Faculty of Medicine and Health Sciences, University of Alcalá, 28801 Alcala de Henares, Spain; 6 Immune System Diseases-Rheumatology and Internal Medicine Service, University Hospital Prince of Asturias, Networking Research Center on for Liver and Digestive Diseases (CIBEREHD), 28806 Alcala de Henares, Spain; 7 Pathological Anatomy Service, University Hospital Gómez-Ulla, 28806 Alcala de Henares, Spain

**Keywords:** chronic venous disease (CVD), circulating cytokines/chemokines, multiplex

## Abstract

**Background:** Chronic venous disease (CVD) comprises a set of vascular disorders that affect the venous system with important local and systemic repercussions. A growing body of evidence displays the relationship between suffering from CVD and a marked deregulation of the immune inflammatory system. In this sense, the previous literature has reported some significant changes in the level of various circulating inflammatory parameters in these patients. However, more research is required to detail and deepen this complex relationship. **Methods:** In this work, we studied, using a multiplex technique, the levels of circulating cytokines and chemokines detectable in the serum of 40 patients with CVD and compared it with 38 healthy controls (HCs). In parallel, we performed Spearman’s correlation analysis to explore potential inflammatory networks in CVD. **Results:** In this study, we measured circulating cytokines and chemokines in CVD patients using a multiplex assay. Results showed increased levels of several pro-inflammatory mediators (IL-1β, IL-2, IL-5, IL-6, IL-7, IL-8, IL-12, IL-17A, IL-23, TNF-α, IFN-γ, fractalkine, ITAC, and GM-CSF) and a decrease in IL-13, with no significant changes in IL-4, IL-10, IL-21, MIP-1α, MIP-1β, or MIP-3α. The Spearman correlation analysis revealed strong, positive correlations among several inflammatory mediators in HC, particularly between TNF-alpha, IL-1β, IL-17A, and IL-23, forming a highly interconnected cytokine network. In contrast, CVD patients showed fewer, weaker, and distinct correlations, with new associations such as IFN-γ with IL-1β and IL-23, suggesting a disrupted inflammatory profile. **Conclusions:** The distinct inflammatory profile in CVD patients, characterized by altered cytokine and chemokine levels and a less coordinated cytokine network, underscores the reconfiguration of inflammatory pathways in this condition. These findings highlight potential therapeutic targets aimed at restoring immune balance and mitigating chronic inflammation in CVD.

## 1. Introduction

Chronic venous disease (CVD) is a term that encompasses an array of vascular disorders affecting the venous system, generally located in the lower limbs [1]. According to epidemiological data, between 60 and 80% of the global population suffers from CVD, with different risk factors recognized such as aging, female sex, obesity, family history, smoking, pregnancy, sedentary behavior, and prolonged standing, among other factors [2,3,4,5,6]. CVD is clinically defined by the presence of venous hypertension and different symptoms and signs of varied severity. Approximately 1 in 4 patients manifest CVD in the form of varicose veins (VVs), and together with reticular veins and telangiectasias, they represent the most common manifestations of CVD [7]. Advanced stages of CVD, accounting for around 5% of total cases, are classified as chronic venous insufficiency (CVI), with severe manifestations that include edemas, skin changes, and ulcerations, which significantly impact the patients’ quality of life and psychological well-being due to aesthetic concerns and long-term complications [8].

From a pathophysiological perspective, both genetic and environmental factors are responsible for inducing structural and functional changes in the venous wall and valves that explain venous hypertension [9,10]. In turn, this elevated venous pressure represents significant stress for the different layers and cells from the venous tissue, inducing further changes and creating a continuous feedback loop that explains the progression of CVD from early stages to CVI [11]. Leukocyte infiltration and inflammation in the venous wall appear as a consequence of venous hypertension-associated damage such as endothelial and microcirculatory dysfunction, edema, and tissue hypoxia [12]. In parallel, exacerbated inflammation enhances the damage in the venous wall and contributes to its remodeling, thus playing a crucial role in the pathogenesis and progression of CVD [13].

Compelling evidence supports that CVD not only has local but also systemic consequences. For instance, previous works have shown that CVD is associated with altered levels of circulating parameters, oxidative stress markers, non-coding RNAs, and different inflammatory mediators [12,14,15]. This systemic inflammation can amplify local venous pathology by perpetuating endothelial cell activation, increasing vascular permeability, and disrupting repair mechanisms. Additionally, the interplay between systemic inflammation and localized hypoxia further drives venous wall stiffening and valve dysfunction, underscoring a vicious cycle of injury and inflammation that is central to the pathogenesis of CVD [12]. Cytokines and chemokines are pivotal players involved in the regulation of immune inflammatory responses and exert significant actions on different tissues and organs [16,17]. An altered production and release of cytokines and chemokines are associated with several diseases with inflammatory bases [18]. In the event of CVD, significant alterations in the serum of different cytokines and chemokines have been reported, also playing a crucial role in the pathogenesis of CVD [19,20]. However, several research gaps remain in understanding the role of cytokines and chemokines in CVD. For example, the relationship of these mediators with different stages of the disease, their interrelation with each other, or with other pathogenic mechanisms associated with CVD are some issues that still need to be addressed.

In the present work, we aim to delve into the study of a broad spectrum of circulating cytokines and chemokines in patients with CVD and compare them with the levels observed in healthy controls (HCs). To achieve this goal, we will exclude patients with CVI or any further comorbidity in order to explore the associations between CVD and inflammatory cytokines and chemokines. A multiplex technique will be used to analyze the levels of 21 circulating cytokines and chemokines detectable in the serum of 49 patients with CVD and 27 HCs. Afterwards, through a statistical analysis, the correlation between studied cytokines and chemokines will be performed.

## 2. Patients and Methods

### 2.1. Study Design

The study was conducted in accordance with fundamental ethical principles, including autonomy, non-maleficence, beneficence, and distributive justice, and adhered to the standards of Good Clinical Practice as well as the principles outlined in the Declaration of Helsinki (2013) and the Oviedo Convention (1997). All collected data complied with current data protection regulations (Organic Law 3/5 December 2018 on the Protection of Personal Data and the Guarantee of Digital Rights, and Regulation (EU) 2016/679). The study protocol was approved by the Clinical Research Ethics Committee of the Gómez-Ulla-UAH Defence Hospital (37/17), and all participants provided written informed consent.

In this cross-sectional study, we examined plasma samples from the cubital vein from 40 patients with a clinical diagnosis of chronic venous disease (CVeD) and compared them with 38 individuals without a history of CVeD (healthy controls, HCs). The Classification System for Chronic Venous Disorders (CEAP), based on clinical, etiological, anatomical, and pathophysiological data, was applied to diagnose CVeD [21]. All included patients were diagnosed with a clinical CEAP state of C1 or C2, which include telangiectasias/reticular veins and VVs, respectively. We did not include patients with CVI, as the clinical manifestations following CEAP are C ≥ 3. The median age of selected patients was 46 [44–55] years old, whereas for HC, it was 55.0 [45.5–59] years old. A total of 27 CVD patients (65.85%) and 21 HCs (55.26%) were women.

The inclusion criteria were as follows: male and female patients diagnosed with CVeD, body mass index (BMI) ≤ 25 kg/m², with or without venous reflux in the great saphenous vein, and a willingness to participate in the follow-up during both preoperative and postoperative phases, as well as to provide tissue and plasma samples. Exclusion criteria included patients without access to their clinical history, those with medical conditions affecting the cardiovascular system (e.g., infections, diabetes, hypertension, or dyslipidemia or thrombosis), venous malformations, arterial insufficiency, substance abuse, or uncertainty regarding their continued participation in the follow-up. The clinical diagnosis of CVeD and venous reflux assessment were performed using non-invasive color Doppler ultrasound (7.5–10 MHz) of the superficial and deep vein systems prior to venous extraction. HCs were carefully chosen to match the patient group in age and sex while ensuring the absence of known inflammatory or cardiovascular conditions, including CVD. Additionally, HCs were required to have a BMI ≤ 25 kg/m² and no history of chronic illnesses, medication use that could influence inflammatory markers, or recent infections. As our main purpose is to explore as precisely as possible the consequences of CVD in circulating cytokines and chemokines, all these inclusion and exclusion criteria aim to control potential confounder variables that might affect their levels in both CVD and HCs. In Table 1, we summarize the main demographic and clinical features of CVD patients and HCs.

### 2.2. Cytokine Determination

In this study, we measured plasma concentrations of the inflammatory cytokines Interleukin 1β (IL-1β), IL-6, IL-12p70, IL-17A, IL-23, tumor necrosis factor alpha (TNFα), and interferon gamma (IFN-γ); chemokines Interferon-inducible T-Cell Alpha Chemoattractant (ITAC), fractalkine (CX3CL1), IL-8 (CXCL8), Macrophage Inflammatory Protein (MIP)-1α, MIP-1β, and MIP-3α; anti-inflammatory cytokines IL-4, IL-10, and IL-13; and cytokines related to immune cell growth and differentiation—granulocyte–macrophage colony-stimulating factor (GM-CSF), IL-2, IL-5, IL-7, and IL-21. For this purpose, aliquots of plasma samples previously obtained from peripheral blood through centrifugation at 2000 rpm for 20 min in a dry tube were used. The samples were stored at −80 °C until the quantification process.

The analysis was performed using the Luminex technique, utilizing a high-sensitivity kit (Milliplex MAP kit, HSTCMAG-28SK-21) from the Merck laboratory (Darmstadt, Germany). In this method, microspheres encoded with specific proportions of red and infrared dye corresponding to the target analytes were used. The microspheres were incubated for 16–18 h with the antigen, allowing binding to the capture antibodies on each microsphere in 96-well plates. After the incubation period, a biotinylated detection antibody specific to each cytokine was added. Finally, a streptavidin–phycoerythrin complex (Strep-PE) was applied, which bound to the detection antibody. The plate was then read using the MAGpix system (Merck). Cytokine concentrations were calculated based on the standard curve, with the analysis performed using Merck analysis program (Analyst). Detection limits for each cytokine were determined in accordance with the protocol specifications.

### 2.3. Statistical Analysis

The software employed was Rstudio 2023.12.1 built 402.

#### 2.3.1. Descriptive Section

Numerical data from all the protein markers obtained through the multiplex assay were included in the descriptive analysis, represented by the mean, standard deviation (sd), median, and interquartile range. The non-parametric Mann–Whitney U test was addressed to assess the statistical significance among groups of study (HCs and CVD). We represented in the body of the article the boxplots corresponding to those values for each protein and in the Supplementary Materials, we detail them through a table.

#### 2.3.2. Correlation Analysis

To assess the relations of collinearity among the protein markers, we performed the correlation analysis with a Spearman matrix correlation as a non-parametric analysis. The elected graphic representation was a pairwise heatmap matrix with the Spearman coefficients, where we exposed in cyan color the directly related variables (positive values), and, on the contrary, the inversely related variables were exposed in salmon color (negative values). Next, the *p*-values were adjusted through the False Discovery Rate correction to diminish bias and are represented as well, according to the level of significance.

## 3. Results

### 3.1. Serum of Patients with Chronic Venous Disease Exhibit Increased Levels of Pro-Inflammatory Cytokines

**Figure 1:**
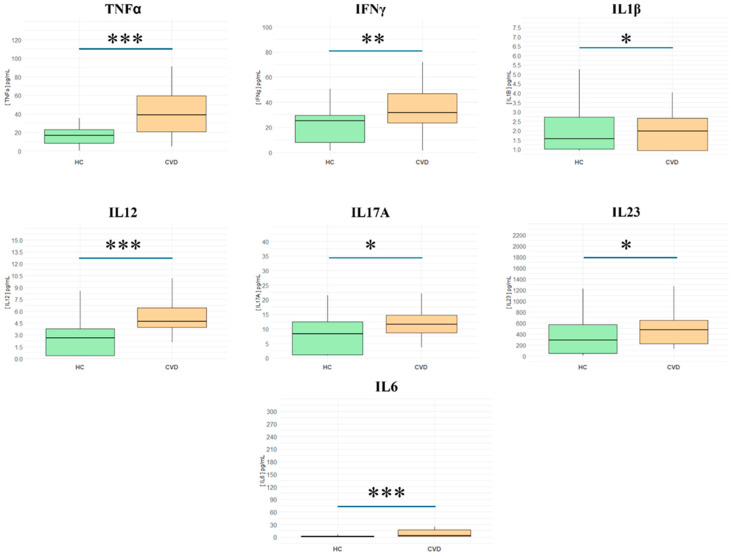
Plasma levels of pro-inflammatory cytokines in healthy controls (HCs) and chronic venous disease patients (CVD). *** *p* < 0.001; ** *p* < 0.01; * *p* < 0.05.

Firstly, the pro-inflammatory cytokines analyzed in this study include TNF-alpha, IFN-gamma, IL-1β, IL-12, IL-17A, IL-23, and IL-6, Supplementary Table S1. As represented in Figure 1, patients with CVD displayed elevated levels compared to controls of **TNF-α** (HCs = 16.83 [8.32–23.1] pg/mL vs. CVD = 38.76 [20.7–59.4] pg/mL; *** *p* < 0.001); **IFN-γ** (HCs = 25.12 [7.90–29.5] pg/mL vs. CVD = 31.51 [23.4–46.9] pg/mL; ** *p* < 0.01); **IL-1β** (HCs = 1.56 [1.01–2.72] pg/mL vs. CVD = 1.98 [0.93–2.66] pg/mL; * *p* < 0.05); **IL-12** (HCs = 2.62 [0.37–3.77] pg/mL vs. CVD = 4.69 [3.94–6.42] pg/mL; *** *p* < 0.001); **IL-17A** (HCs = 8.33 [1.16–12.4] pg/mL vs. CVD = 11.54 [8.72–14.8] pg/mL; * *p* < 0.05); **IL-23** (HCs = 292.88 [48.6–571] pg/mL vs. CVD = 473.39 [224–652] pg/mL; * *p* < 0.05); and **IL-6 levels** (HCs = 1.05 [0.36–2.94] pg/mL vs. CVD = 3.7 [1.48–17.2] pg/mL; *** *p* < 0.001).

### 3.2. Serum of Patients with Chronic Venous Disease Displays a Marked Decreased Level of the Anti-Inflammatory Cytokine IL-13

**Figure 2:**
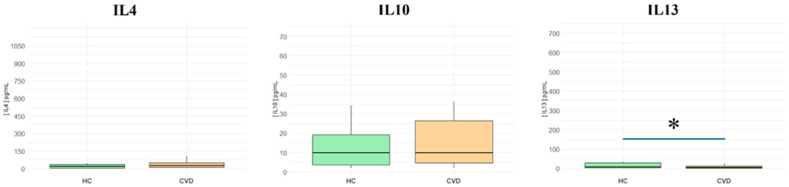
Plasma levels of anti-inflammatory cytokines in healthy controls (HCs) and chronic venous disease patients (CVD). * *p* < 0.05.

The levels of the anti-inflammatory cytokines IL-4, IL-10, and IL-13 were also measured (Supplementary Table S1). As represented in Figure 2, while there was a notable decrease in **IL-13** (HCs = 7.55 [2.51–28.9] pg/mL vs. CVD = 2.95 [0.53–12.3] pg/mL; * *p* < 0.05), there were no significant changes observed in **IL-4** (HCs = 19.18 [5.4–33.8] pg/mL vs. CVD = 25.66 [10.4–49] pg/mL; NS); or IL-10 (HCs = 9.86 [3.55–19.1] pg/mL vs. CVD = 9.87 [4.62–26.4] pg/mL; NS) levels between the two groups 

### 3.3. Serum of Patients with Chronic Venous Disease Exhibits Raised Levels of Cytokines Related to Immune Cell Growth and Differentiation

**Figure 3:**
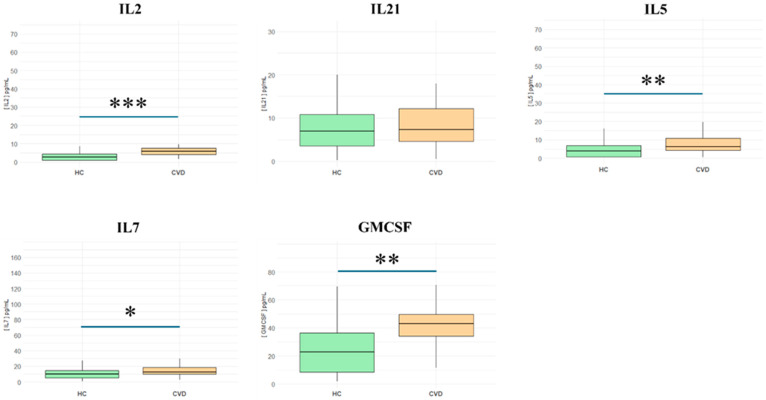
Plasma levels of immunomodulatory cytokines and growth factors in healthy controls (HCs) and chronic venous disease patients (CVD). *** *p* < 0.001; ** *p* < 0.01; * *p* < 0.05.

This study included immunomodulatory cytokines such as IL-2, IL-21, IL-5, IL-7, and GM-CSF. As represented in Figure 3, in patients with CVD, we observed a significant increase in plasma levels of **IL-2** (HCs = 2.59 [0.9–4.46] pg/mL vs. CVD = 5.9 [4.1–7.6] pg/mL; *** *p* < 0.001); **IL-5** (HCs = 3.94 [0.695–6.92] pg/mL vs. CVD = 6.28 [4.41–10.9] pg/mL; ** *p* < 0.01); **IL-7** (HCs = 9.99 [4.95–14.6] pg/mL vs. CVD = 12.54 [10.1–18.4] pg/mL; *** *p* < 0.001); and **GM-CSF** (HCs = 22.93 [8.58–36.4] pg/mL vs. CVD = 43.11 [34.3–49.5] pg/mL; ** *p* < 0.01). However, no significant differences were observed for **IL-21** (HCs = 6.99 [3.52–10.8] pg/mL vs. CVD = 7.3 [4.67–12.1] pg/mL; NS).

### 3.4. Serum of Patients with Chronic Venous Disease Exhibits Augmented Levels of the Chemokines IL-8, Fractalkine, and ITAC

**Figure 4:**
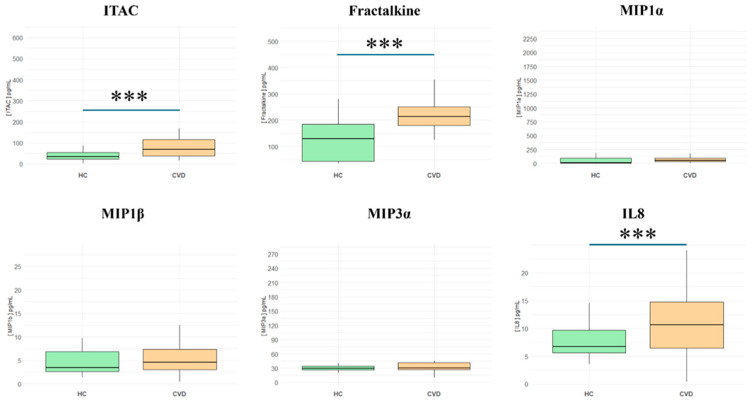
Plasma levels of chemokines in healthy controls (HCs) and chronic venous disease patients (CVD). *** *p* < 0.001.

Chemokines analyzed in this study include ITAC, fractalkine, MIP-1α, MIP-1β, MIP-3α, and IL-8 (Supplementary Table S1). As represented in Figure 4, we report increased levels of **ITAC** in CVD patients (HCs = 34.77 [22.9–53.4] pg/mL vs. CVD = 69.28 [38.3–115] pg/mL; *** *p* < 0.001); **fractalkine** (HCs = 128.85 [43.3–184] pg/mL vs. CVD = 212.75 [180–251] pg/mL; *** *p* < 0.001); and **IL-8** (HCs = 6.76 [5.62–9.69] pg/mL vs. CVD = 10.66 [6.42–14.8] pg/mL; *** *p* < 0.001); meanwhile, there were no significant differences in **MIP-1α** (HCs = 9.4 [5.08–90] pg/mL vs. CVD = 48.7 [29.4–91.1] pg/mL; NS); **MIP-1β** (HCs = 3.43 [2.65–6.86] pg/mL vs. CVD = 4.58 [3.01–7.33] pg/mL; NS); and **MIP-3α** (HCs = 29.7 [26.2–34] pg/mL vs. CVD = 30.59 [27.1–41.5] pg/mL; NS).

### 3.5. Comparative Correlation Analysis of Inflammatory Cytokines and Chemokines in Chronic Venous Disease Patients and Healthy Controls

**Figure 5:**
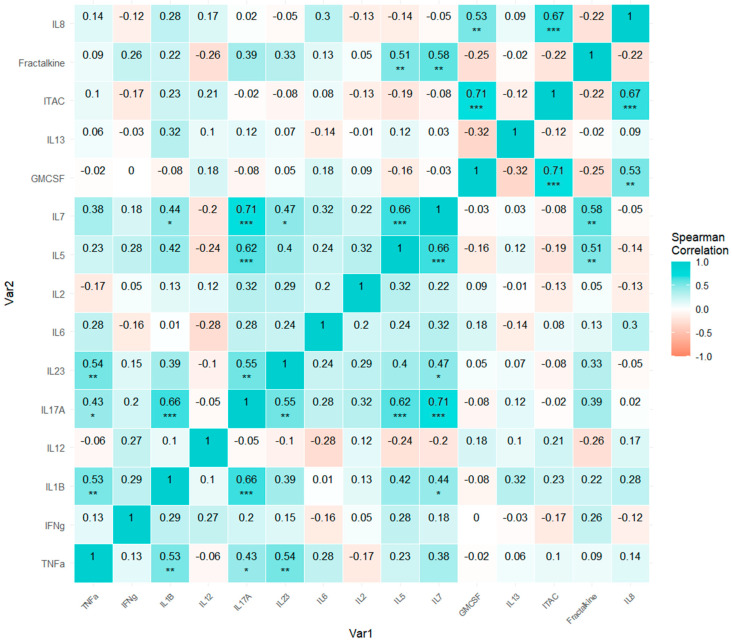
A pairwise heatmap matrix with Spearman correlation coefficients for the healthy control (HC) subjects. *p*-Values associated with each Spearman correlation coefficient appear with asterisks and are adjusted by False Discovery Rate correction (FDR): adjusted alpha (*** < 0.001, ** < 0.01, * < 0.05)—*** (0.00004, 0.00057); ** (0.00173, 0.00954); * (0.02396, 0.04813). Positive or direct association is represented through a cyan color whereas negative or inverse association is represented through a salmon color, with values represented with a minus (-) sign.

A Spearman correlation analysis was performed to examine the relationships between circulating cytokines and chemokines in CVD patients and HCs. In the HCs (Figure 5), we identified multiple significant positive correlations among various cytokines and chemokines. TNF-alpha showed correlations with IL-1β, IL-17A, and IL-23. IL-1β correlated with IL-17A, IL-5, and IL-7, and IL-17A correlated with IL-23, IL-5, and IL-7. IL-23 was additionally correlated with IL-7, while IL-5 displayed correlations with both IL-7 and fractalkine. IL-7 was further correlated with fractalkine. The immunomodulatory cytokine GM-CSF correlated with both ITAC and IL-8, and ITAC also showed a correlation with IL-8.

**Figure 6:**
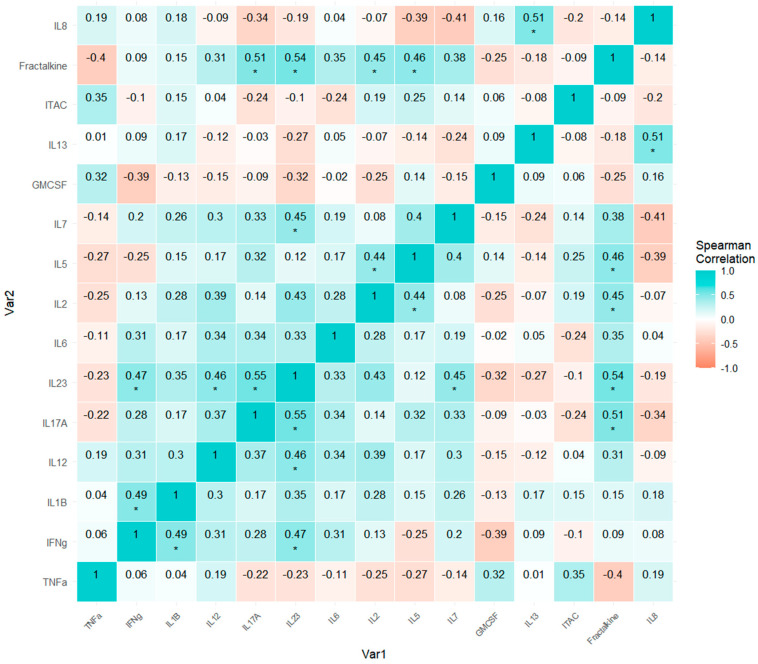
A pairwise heatmap matrix with Spearman correlation coefficients for the chronic venous disease (CVD) subjects. *p*-Values associated with each Spearman correlation coefficient appear with asterisks and are adjusted by False Discovery Rate correction (FDR): adjusted alpha (* < 0.05)—* (0.01780, 0.0430). Positive or direct association is represented through a cyan color whereas negative or inverse association is represented through a salmon color.

In the CVD patient group (Figure 6), fewer significant correlations were observed, and those present were generally weaker (marked by a single *). The correlation patterns also differed from those in controls. IFN-gamma showed correlations with IL-1β and IL-23, IL-12 with IL-23, and IL-17A with IL-23 and fractalkine. IL-2 correlated with IL-5 and fractalkine, while IL-23 was correlated with IL-7 and fractalkine. IL-5 was also correlated with fractalkine, and IL-8 displayed a correlation with IL-13.

These findings indicate a different pattern of cytokine and chemokine interactions between CVD patients and healthy controls, with notable differences in the number, strength, and specific pairs of correlated inflammatory mediators.

## 4. Discussion

In this study, we assessed circulating levels of cytokines and chemokines in patients with CVD using a multiplex assay, revealing elevated levels of several pro-inflammatory mediators (including IL-1β, IL-2, IL-5, IL-6, IL-7, IL-8, IL-12, IL-17A, IL-23, TNF-α, IFN-γ, fractalkine, ITAC, and GM-CSF) and a decrease in IL-13. No significant differences were observed for L-4, IL-10, IL-21, MIP-1α, MIP-1β, and MIP-3α. Spearman’s correlation analysis identified significant positive correlations among these inflammatory mediators, indicating potential interactions and concerted upregulation pathways in CVD. Specifically, we observed interconnected positive correlations among TNF-α, IL-1β, IL-17A, IL-23, IL-21, IL-5, IL-7, and fractalkine, suggesting a network of inflammation. GM-CSF was associated with ITAC and IL-8, while ITAC further correlated with IL-8, indicating additional inflammatory axes. For providing greater clarity, the present discussion will be divided into three subsections.

### 4.1. Inflammatory Mediator Profiles in CVD: Insights from Previous Research and Comparative Contexts

Past works have highlighted a pro-inflammatory switch in the levels of circulating cytokines and chemokines of patients with CVD, highlighting their importance in the inflammatory processes and pathophysiological mechanisms associated with the disease. For instance, elevated levels of inflammatory markers like IL-6, matrix metalloproteinases (MMPs), and adhesion molecules such as VCAM-1 and ICAM-1 have been consistently reported in patients with CVD [22,23]. According to the literature [12], circulating cytokines and other substances not only reflect the inflammatory state but also contribute to vascular remodeling and the progression of CVD. Therefore, the altered levels of cytokines observed in our study are potentially associated with the different changes affecting the venous wall in these subjects.

In parallel, studies have shown interesting differences depending on the region where the blood was drawn. Grudzińska et al. [19] quantified the levels of cytokines with and without stimulation by phytohemagglutinin (PHA) released by lymphocytes in 34 patients with CVD obtained from the great saphenous vein (GSV) and cubital vein and compared it with 12 HCs. They observed that in both stimulated and unstimulated samples, lymphocytes from patients with CVD presented higher IL-1β and IFN-γ concentrations with a marked reduction in CCL5, whereas IL-4 and IL-17A had higher concentrations without stimulation and TNF-α had higher concentrations with stimulation when compared to the HCs. Also, they recognized a distinct cytokine profile in the CVD group when the blood was extracted from the GSV in comparison to the cubital vein, suggesting that the oscillatory flow present in incompetent veins causes changes in the cytokine production by lymphocytes, promoting a pro-inflammatory switch. Similarly, Lattimer et al. [20] analyzed through a multiplex technique the levels of different cytokines and chemokines in plasma samples drawn from the arms and legs of 24 patients with CVD and 24 controls. They observed that within the patients, blood extracted from lower limbs showed increased levels of IL-6, IL-8, and MCP-1 when compared to that extracted from the arm. This geographical variability in sampling may have influenced the comparison with healthy controls in our study. As the blood samples in our research were taken from the cubital vein, the results likely reflect systemic, rather than localized, inflammatory changes associated with CVD. While this approach enables comparison with other systemic studies, it may not capture the heightened local inflammatory response occurring in the lower limbs of CVD patients. This could partly explain why some pro-inflammatory cytokines, such as IL-4, IL-10, IL-21, MIP-1α, MIP-1β, and MIP-3α, did not show significant variations. Future research incorporating blood samples from both systemic and localized regions, such as the GSV, may provide deeper insights into the spatial dynamics of cytokine production in CVD.

The study of circulating cytokines in the context of CVD opens avenues for exploring targeted therapeutic interventions. For example, sulodexide, a glycosaminoglycan used to treat CVD, has demonstrated the capacity to reduce the levels of several pro-inflammatory markers, such as IL-6 and TNF-α, indicating that targeting these cytokines and chemokines could be a viable therapeutic approach to manage CVD progression [24]. This anti-inflammatory effect of sulodexide suggests its potential not only to alleviate symptoms but also to modulate the chronic inflammatory processes underlying CVD. Similarly, compression therapy has been shown to significantly decrease the levels of IL-1β, IL-6, IL-8, IL-12p40, G-CSF, GM-CSF, IFN-γ, TNF-α, and other inflammatory mediators when compared to untreated patients [23]. This highlights the role of mechanical interventions in reducing venous stasis and inflammation. The anti-inflammatory effects of compression therapy may be linked to improved venous return, reduced endothelial activation, and a decrease in leukocyte adhesion. Similarly, Tisato et al. [25] observed the effects of a saphenous sparing surgical correction (CHIVA strategy) in 32 patients affected by CVI with superficial venous reflux. They found that elevated levels of pro-inflammatory cytokines like IL-8, PDGF, EGF, VEGF, and RANTES were observed shortly after CHIVA surgery, reflecting endothelial remodeling, but these levels significantly decreased by six months post-treatment, often approaching those of healthy individuals. Exceptions included MCP-1, G-CSF, and CXCL10. Notably, patients with disease relapse showed persistently high levels of certain cytokines, suggesting their potential as biomarkers for disease progression and recurrence (EGF, PDGF, and RANTES). Future therapeutic strategies could involve a combination of pharmacological, surgical, and mechanical treatments tailored to individual inflammatory profiles. Likewise, inflammatory mediators like TNF-α could represent potential therapeutic targets for certain patients with advanced stages of CVD, as found in a pilot study [26]. Further studies are warranted to explore the effects of potential immunomodulatory approaches in CVD patients, being guided by their cytokine and chemokine profile.

Intra-individual variation in cytokine and chemokine levels in patients with CVD can be influenced by a multitude of factors. For instance, Franklin et al. [27] reported that the disease stage seems to represent a critical variable to be considered in these variations. In more detail, they report that patients with VVs without leg ulcers present an increased detection of inflammatory cytokines (IL-2, IL-12, IFN-γ) when compared to HCs and patients with leg ulcers (C5-C6), claiming that advanced CVD stages are potentially associated with an exhausted immune state associated with T-cell dysfunction in the setting of chronic wounds. Similar observations were made by Guss et al. [28], suggesting that disease stages could potentially explain intra-individual cytokine and chemokine levels. Likewise, the presence of systemic conditions (i.e., obesity), genetic and environmental triggers (i.e., infections), aging, and even seasonal variations in cytokines and chemokines have also been found in past works [29,30,31,32,33,34,35]. These findings underscore the importance of considering cytokine and chemokine profiling not only at a single time point but also longitudinally and integrating or controlling all these variables to better understand the inflammatory milieu and guide personalized treatments. Likewise, the reproducibility of cytokine and chemokine levels according to these factors should be explored more deeply in future works, with other components potentially affecting these variations like demographics, lifestyle, and technical aspects of measurement being considered.

Similarly, it is also interesting to compare our results with different manifestations of CVD such as gestational venous hypertension (GVH). GVH develops because of hemodynamic, hormonal, and mechanical changes associated with pregnancy, causing an impairment in venous return from the lower limbs to the heart [36]. In this sense, we demonstrated a pro-inflammatory profile in the blood extracted from the arms and umbilical cord of pregnant women with CVD and their newborns, respectively. Specifically, we observed a marked increase in pro-inflammatory mediators (IL-6, TNF-α, IL-2, IL-8, IL-12, IL-17, IL-21, IL-23, CX3CL1, CCL4, CCL20, IL-5, GM-CSF, MIP-1b, MIP-3a) along with a reduction in the anti-inflammatory mediators IL-4, IL-10, and IL-13, as well as in interferon-gamma (IFN-γ) [37]. It is important to compare the results reported in the work with those obtained in the present study, as patients with non-gestational CVD also share a marked increase in some pro-inflammatory mediators (IL-2, IL-5, IL-6, IL-7, IL-8, IL-12, IL-23, TNF-α, IFN-γ, GM-CSF, and CX3CL1) and a decrease in IL-13, whereas other cytokines dysregulated in GVH appear to be unaltered in our study like IL-4, IL-10, IL-21, MIP-3α, and MIP-1β. ITAC is differentially altered in CVD but not GVH patients. Pregnancy itself is mostly an inflammatory condition [38] and a broad spectrum of research supports the pathogenic role of GVH in maternofetal structures like the placenta and umbilical cord [36]. Therefore, it is likely that the shared cytokines and chemokines observed in our study could be more related to proper CVD, whereas the non-coincident cytokines may be more related to pregnancy and the consequences of CVD in this period. However, future studies in these groups should be performed to confirm this hypothesis.

### 4.2. Potential Pathogenic Role and Relevance of Altered Cytokines and Chemokines in CVD

Firstly, the elevated levels of classic pro-inflammatory cytokines such as IL-1β, TNF-α, and IL-6 underscore a strong inflammatory response associated with CVD. An increased detection of these cytokines has been associated with the development and progression of major pathogenic mechanisms of CVD such as hemodynamic changes, hypoxia, vascular matrix remodeling and injury, oxidative stress, leukocyte recruitment, and endothelial dysfunction [39]. Polymorphisms in the IL-1β, IL-6, and TNF-α genes have been associated with an increased risk and severity of CVD, particularly influencing factors such as ulceration, age of disease onset, and comorbid conditions like arterial hypertension and ischemic heart disease. These genetic variations may serve as potential biomarkers for identifying individuals at higher risk of severe CVD progression [40].

IL-1β and TNF-α are central mediators in the inflammatory cascade, often exerting synergic effects capable of inducing vascular endothelial activation and increasing the permeability of vascular walls, promoting the expression of adhesion molecules such as ICAM-1 and VCAM-1, thereby facilitating immune cell infiltration [41]. IL-1β is a cytokine associated with NLRP3 inflammasome activation [42]. Recently, Gou et al. [43] demonstrated that plasma levels of TNF-α, IL-1β, and NLRP3 inflammasomes could be potentially used as risk factors for venous ulcers in patients with VVs of the lower extremity. Additionally, IL-1β and TNF-α have been shown to stimulate the production of reactive oxygen species (ROS) and matrix metalloproteinases (MMPs), leading to extracellular matrix degradation and vascular remodeling, processes that are pivotal in the development and progression of CVD [44,45,46]. Likewise, IL-1β and TNF-α are implicated in the production of various inflammatory cytokines and chemokines, either dependently or independently of the activation of transcriptional factors, such as NF-ΚB, enhancing endothelial cell dysfunction, vascular inflammation, and apoptosis [44,47]. IL-6 induces the expression of a broad spectrum of proteins responsible for acute inflammation by hepatocytes and directs leukocyte trafficking and activation. This cytokine also exerts important functions in adaptative immunity, by promoting T-cell proliferation, B-cell differentiation and survival, and the plasma-cell production of IgG, IgA, and IgM [48]. Increased IL-6 expression has been seen in the plasma of patients with a venous thromboembolism, which may play a vital role in inflammatory injury of vascular endothelial cells [49]. Past works have found that CVD, and specifically CVI, is a condition associated with a venous thromboembolism [50], and IL-6 could be a potential link between those entities to be explored in future works. Moreover, IL-6 can amplify the hypoxic responses in venous tissues by upregulating hypoxia-inducible factor 1-alpha (HIF-1α) [51], which exacerbates venous wall remodeling and dysfunction. By amplifying local and systemic inflammatory responses, IL-1β, TNF-α, and IL-6 emerge as critical targets for developing therapies aimed at mitigating both the inflammatory and structural progression of CVD.

The increased levels of IL-17A and IL-23 suggest the activation of the Th17 pathway, which is implicated in chronic inflammatory and autoimmune diseases [52]. Both IL-17A and IL-23 have been associated with host immune defenses, tissue repair, and inflammatory disease pathogenesis. IL-17A, produced primarily by Th17 cells, drives inflammation by inducing the release of other pro-inflammatory cytokines and by recruiting neutrophils and macrophages [53]. Simultaneously, further studies have demonstrated that IL-17A can directly induce the expression of MMPs [54], which may lead to vascular remodeling and weakening of venous walls in CVD subjects. IL-23 is essential in supporting Th17 cell survival and differentiation, further amplifying the inflammatory response [55]. Additionally, IL-23 has been shown to upregulate the production of inflammatory cytokines such as IL-6 and TNF-α, further promoting inflammation within the venous system [56]. In a study developed in 2022, Amato et al. [57] reported that Th17 markers behave as empirical risk factors for CVD development, supporting the TH17-dependent pathogenesis of CVD. This Th17-related cytokine axis indicates a specialized immune response that may be central to the chronic, non-resolving inflammation seen in CVD, highlighting a pathway potentially responsible for sustained immune cell activity and tissue damage in the venous walls.

IL-8 (also known as CXCL8), CX3CL1 (or fractalkine), and ITAC (CXCL11) are three major chemokines involved in leukocyte trafficking. IL-8 is primarily involved in neutrophil migration, while fractalkine facilitates NK, monocyte, and T-cell trafficking [58]. ITAC, associated with Th1 responses, attracts activated T cells and macrophages [59]. Altered levels of these chemokines have been implicated in various vascular diseases, by promoting inflammation and immune cell recruitment, which drives disease progression [60,61]. An increased expression of IL-8 in the venous wall has been observed in patients with VVs as well as in the blood of affected individuals, suggesting its role in exacerbating venous inflammation and remodeling [20,62]. While research on the roles of fractalkine and ITAC in CVD is more limited, fractalkine has been shown to mediate monocyte migration within vascular tissues, and blocking its receptor (CX3CR1) can prevent monocyte and macrophage accumulation, offering potential therapeutic targets [63]. Similarly, ITAC, along with related chemokines like CXCL9 and CXCL10, participates in inflammatory pathways that recruit immune cells to vascular lesions [64], potentially contributing to CVD pathogenesis. Given these findings, further research into the roles of IL-8, fractalkine, and ITAC in CVD is crucial. Understanding their contributions could lead to novel therapeutic strategies aimed at modulating these chemokines to reduce inflammation and mitigate the progression of this condition.

IL-5 is a crucial factor for B-cell growth, differentiation, and the selection of immunoglobulin A (IgA). In conjunction with granulocyte–macrophage colony-stimulating factor (GM-CSF), IL-5 is instrumental in the function and development of eosinophils, often referred to as “eosinopoietins” [65]. Research has demonstrated the synergistic effects of GM-CSF and IL-5 on eosinophil activation during inflammatory diseases [66]. The synergy between IL-5 and GM-CSF in CVD could lead to a potent activation of eosinophils within the affected venous tissue. GM-CSF activates eosinophils to produce various chemokines and cytokines, such as IL-1, IL-6, and TNF-α, which further attract immune cells like T cells to the site of inflammation. This influx intensifies the inflammatory response, creating a feedback loop that exacerbates tissue damage and perpetuates a state of chronic inflammation, which is characteristic of CVD [67,68]. Moreover, the interaction between IL-5 and GM-CSF can upregulate the expression of adhesion molecules on endothelial cells, facilitating the adhesion and transmigration of eosinophils and other leukocytes into the venous intima, perpetuating inflammation and tissue damage [69]. Contrary to our results, reduced levels of IL-5 were found in lymphocytes from patients with great saphenous vein incompetence [70]. Decreased levels of GM-CSF are also reported in the serum of patients with CVI [71], and in vitro studies have shown that GM-CSF may play an important role on endothelial cells by inducing their proliferation and counteracting induction intercellular adhesion molecule (ICAM)-1, vascular cell adhesion molecule (VCAM)-1, and osteoprotegerin (OPG) expression upon exposure to TNF-α [72], suggesting a possible protective role of this cytokine. Overall, we hypothesize that increased levels of IL-5 and GM-CSF could signify a pro-inflammatory state that supports eosinophilic inflammation and tissue remodeling within venous structures in earlier stages of CVD. This combination may thus worsen the pathological changes in venous walls by maintaining an environment of chronic inflammation and cellular damage, suggesting that both IL-5 and GM-CSF might be valuable targets for therapeutic intervention in CVD to reduce inflammation and slow disease progression.

IL-2 and IL-7 are key cytokines involved in proliferative cell divisions and immune regulation, often exerting synergistic effects [73]. IL-2, produced by activated T cells, plays a critical role in expanding T-cell populations and maintaining immune balance. It also suppresses excessive immune activation, preventing autoimmunity, and is essential for the development of Treg cells [74]. On the other hand, IL-7, mainly produced by stromal and epithelial cells, is essential for immune cell development and homeostasis, especially for T and B lymphocytes, as well as NK cells [75,76]. In the context of CVD, IL-2 may influence inflammatory pathways, leading to vascular dysfunction, including endothelial permeability and leukocyte adhesion, which are key features of vascular diseases like atherosclerosis [77]. IL-7 has also been identified as a significant mediator in vascular inflammation, promoting the recruitment of monocytes and macrophages to the endothelium, a process implicated in various vascular disorders [78,79]. Thus, IL-2 and IL-7 could synergistically contribute to a pro-inflammatory environment in CVD by amplifying T-cell responses and supporting macrophage recruitment to affected vascular regions. This could worsen tissue damage, promoting chronic inflammation and advancing the disease. Other works exploring this cytokine found that lymphocytes isolated from patients with great saphenous vein incompetence exhibited increased IL-2 expression without stimulation but reduced IL-2 levels upon stimulation when compared to samples from the upper limbs [19]. IL-7 levels were found to be decreased in patients with CVI [71], although its precise role in early-stage CVD remains to be further explored. Overall, both IL-2 and IL-7 likely contribute to a pro-inflammatory environment, exacerbating immune responses and promoting the progression of CVD. Further research is needed to fully understand their roles and therapeutic potential in managing CVD.

Elevated levels of IFN-γ and IL-12 in serum suggest a sustained activation of the cellular immune response and inflammation, especially through the activation of Th1 responses. IL-12 is crucial for T-cell differentiation into the Th1 phenotype, amplifying this inflammation by promoting further IFN-γ production [80,81]. Increased levels of IL-12 and IFN-γ and exacerbated Th1 responses have been associated with advanced stages of CVD [30,82]. Past works have demonstrated that IFN-γ is able to promote vascular inflammatory response by causing impairment of endothelial barrier function partly through the increase in p38 MAP kinase and reduction in nitric oxide (NO) [83]. The aberrant activation of p38 and other MAP kinases has been observed in the venous wall and fibroblasts of patients with CVD [84,85], whereas altered levels of NO and their regulators like NO synthases (NOSs) have also been reported [15,86]. IFN-γ may be involved in these mechanisms, although further studies are still warranted. Collectively, both IL-12 and IFN-γ are indicative of an exacerbated Th1 response occurring in patients with CVD as well as potential pathogenic mechanisms affecting the endothelium and vascular wall of VVs.

Finally, a reduction in IL-13 levels, the only cytokine decreased in our study, may indicate an impaired anti-inflammatory response, potentially exacerbating chronic inflammation in the affected venous tissues. IL-13, primarily produced by Th2 cells, is known for its role in limiting inflammation and promoting tissue repair and fibrosis [87,88]. IL-13 exerts anti-inflammatory effects by downregulating the production of pro-inflammatory cytokines such as IL-1β, TNF-α, and IL-6, and by reducing macrophage activation and recruitment [89]. Thus, lower levels of IL-13 in CVD patients could suggest a diminished ability to counteract the persistent inflammatory signals typically found in this condition. Likewise, IL-13 can also regulate MMP-2, -9, -12, -13, and -14 along with other components like a u tissue-type plasminogen activator (t-PA) and urokinase-type plasminogen activator (u-PA) from endothelial cells, vascular smooth muscle cells, and monocytes/macrophages [47]. Low plasma levels of IL-13 in CVD patients may impair its regulatory role in matrix remodeling by reducing its ability to induce MMPs in a controlled manner and balance ECM degradation. This deficiency could exacerbate vascular ECM breakdown and disrupt tissue repair processes, contributing to progressive venous wall weakening and chronic inflammation. Therefore, IL-13 reduction may disrupt the balance between pro- and anti-inflammatory pathways, leading to unopposed chronic inflammation, vascular damage, and reduced tissue repair capacity. However, future studies deepening knowledge on the possible implications of the decrease in this cytokine are still warranted.

Overall, in Table 2, we summarize the main cytokines altered in our study, their status in CVD patients, their inflammatory/anti-inflammatory mechanisms, and their potential implications in the event of CVD.

### 4.3. Unaltered Cytokines and Chemokines

Finally, our research failed to find significant alterations in some critical cytokines and chemokines such as IL-4, IL-10, IL-21, MIP-1α, MIP-1β, and MIP-3α.

IL-4 is crucial for the differentiation of naive T-helper cells into Th2 cells and promotes the production of anti-inflammatory cytokines. It also contributes to tissue repair and maintaining physiological balance [87]. Similarly, IL-10 is produced by various immune cells, including T cells, B cells, and macrophages, and serves as a potent suppressor of macrophage and T-cell functions, limiting host responses to antigens [17,90]. IL-21, a pleiotropic cytokine, influences the function of T and B cells and has been implicated in various inflammatory and autoimmune conditions [91].

The lack of significant changes in the levels of these cytokines in our CVD cohort may suggest that the anti-inflammatory pathways mediated by IL-4, IL-10, and IL-21 are not prominently altered in the early stages of CVD (CEAP C1 and C2). This observation aligns with studies indicating that while pro-inflammatory cytokines are often elevated in cardiovascular and inflammatory conditions, anti-inflammatory cytokines like IL-4 and IL-10 may remain unchanged or even decrease, reflecting a potential imbalance between pro- and anti-inflammatory forces [92,93]. Furthermore, the unchanged levels of IL-21 in our study are consistent with findings in other inflammatory diseases where IL-21’s role appears to be more context-dependent, influencing both protective immunity and immunopathology [94].

MIP-1α (CCL3) and MIP-1β (CCL4) are chemokines known to recruit monocytes, lymphocytes, and other immune cells to sites of inflammation. Their roles have been extensively studied in various inflammatory conditions, including viral infections and hematopoiesis [95,96]. MIP-3α (CCL-20) is primarily associated with the recruitment of lymphocytes and dendritic cells and is implicated in mucosal immunity [97]. Their lack of significant alteration in our CVD cohort may indicate that these chemokines are not central to the inflammatory processes driving early-stage CVD (CEAP C1 and C2). This aligns with findings from studies on varicose veins, where certain chemokines were upregulated, while others, including MIP-1α and MIP-1β, did not show marked changes [62].

Overall, unchanged levels of these cytokines and chemokines in our CVD patients suggest that IL-4, IL-10, IL-21, MIP-1α, MIP-1β, and MIP-3α may not be actively engaged in the early inflammatory milieu of CVD. However, further studies should confirm these results and explore their possible implications in other contexts. For instance, IL-10, IL-21, MIP-3α, and MIP-1β were found to be altered in women with CVD during pregnancy and their newborns [37], supporting their potential implications in other subjects affected by this condition.

### 4.4. Interconnections and Correlation Patterns Among Inflammatory Mediators

When comparing the correlation patterns among cytokines, chemokines, and other immunomodulatory factors in HC individuals (Figure 5) and those with CVD (Figure 6), we observe differences in each variable pair regarding both the strength of association (the absolute value of the Spearman coefficient) and the type of relationship (positive/direct or negative/inverse correlation). In HC individuals, certain combinations of factors show positive correlations, whereas in individuals with CVD, these same combinations exhibit negative or null correlations.

We hypothesize that the observed differences in cytokine and chemokine correlations between patients with CVD and HCs indicate a substantial alteration in inflammatory pathway regulation in CVD patients. In the control group, significant, well-structured correlations were found, with key pro-inflammatory mediators like TNF-alpha, IL-1β, IL-17A, and IL-23 showing strong positive associations. This coordinated network in healthy individuals suggests an immune system that responds in a balanced and regulated manner to inflammatory stimuli. The correlation of IL-1β with IL-17A, IL-5, and IL-7, or TNF-alpha with IL-1β, IL-17A, and IL-23, highlights a central role of these cytokines in modulating inflammation in a manner that may maintain homeostasis and prevent excessive immune activation.

In contrast, CVD patients exhibit fewer significant correlations, generally with weaker intensities, and these connections differ substantially from the control group, potentially indicating a dysregulation in the inflammatory signaling network. For example, novel correlations emerge in CVD patients, such as IFN-γ with IL-1β and IL-23, and IL-8 with IL-13. This reorganization may reflect compensatory mechanisms that arise in response to chronic inflammation and endothelial dysfunction characteristic of CVD. The appearance of new correlations, along with the loss of those present in healthy controls (e.g., those involving TNF-α and IL-1β with other cytokines), suggests an adaptive immune response attempting to adjust to persistent inflammatory signaling. This interpretation is consistent with studies showing that chronic inflammation in CVD can alter cytokine and chemokine expression, potentially due to factors like oxidative stress and local tissue hypoxia.

The reduced number and strength of correlations in the CVD group may reflect an impaired ability to coordinate an effective inflammatory response, which could lead to a disorganized immune activation. This shift could result from the adaptive response to chronic inflammation and tissue remodeling typical in CVD, which disrupts intracellular signaling pathways and affects interactions among immune mediators.

In summary, comparing the inflammatory networks between the two groups highlights how CVD profoundly alters mediator interactions. Healthy individuals exhibit a coordinated, robust cytokine network, while CVD patients show a disorganized network with different interactions. These findings suggest that cytokine dysregulation in CVD not only reflects the underlying chronic inflammation but also a reorganization of the inflammatory network in response to chronic tissue damage and repair attempts. This altered network should be considered in developing therapeutic strategies aimed at restoring immune regulation in CVD.

### 4.5. Limitations of Our Study and Future Research Directions

This study has several limitations that should be acknowledged and addressed in future research. Firstly, the cross-sectional nature of the study limits the ability to track changes in inflammatory mediators over time or their progression alongside disease stages. The relatively small sample size may also reduce the statistical power to detect subtle differences or rare correlations. While the study focuses on circulating cytokines and chemokines, it does not explore related mechanisms such as oxidative stress, endothelial dysfunction, or the role of non-coding RNAs, which could provide complementary insights. Likewise, the exclusion of patients with severe chronic venous insufficiency (CEAP ≥ 3) limits the generalizability of our results to early-stage CVD and does not address the inflammatory profiles in advanced disease stages. The inclusion of patients with a BMI ≤ 25 kg/m^2^, while aimed at minimizing confounding effects of obesity on inflammation, restricts the applicability of our findings to the broader CVD population, many of whom have a higher BMI. Additionally, potential confounding factors such as lifestyle variables (e.g., smoking, diet, physical activity) and medication use were not controlled, which may influence the inflammatory profiles observed. Finally, while multiplex assays offer a broad and efficient means to assess inflammatory mediators, they are subject to technical limitations such as cross-reactivity and detection variability, which could impact the precision of the results. Addressing these limitations in future research will help to provide a more comprehensive understanding of the inflammatory mechanisms underlying CVD.

## 5. Conclusions

Our findings demonstrate that patients with chronic venous disease (CVD) exhibit distinct cytokine and chemokine profiles characterized by elevated levels of several pro-inflammatory markers (e.g., IL-1β, IL-2, IL-5, IL-6, IL-7, IL-8, IL-12, IL-17A, IL-23, TNF-α, IFN-γ, fractalkine, ITAC, GM-CSF) and reduced IL-13, compared to healthy controls. This suggests a shift toward a pro-inflammatory environment, potentially driving the chronic inflammation and tissue remodeling associated with CVD. A correlation analysis further revealed a disrupted and reconfigured inflammatory network in CVD patients, with weaker connections and new associations among cytokines, such as IFN-γ correlating with IL-1β and IL-23.

These results highlight the potential of cytokine profiling as a biomarker for CVD severity or progression. However, further studies are needed to assess the specificity and sensitivity of these markers in differentiating between disease stages or monitoring treatment responses. While our findings provide insights into inflammatory mechanisms, the utility of these cytokines as clinical biomarkers will require validation in larger, longitudinal studies to establish their predictive accuracy and therapeutic relevance. 

## Figures and Tables

**Table 1:** Demographic and clinical features of CVD patients included in our study. It should be noted that neither CVD patients nor HCs presented any other chronic diseases or comorbidities, medication use, or recent infections (exclusion criteria). N/A= Not applicable.

	CVD (n = 40)	HCs (n = 38)
Female sex (%)	27 (65.85%)	21 (55.26%)
Median age [Q1–Q3]	46 [44–55]	55.0 [45.5–59]
Body mass index	23.01 ± 1.16	22.97 ± 1.05
Smoking habits Yes No	15 (37.50%)/ 25 (62.50%)	11(28.94%)/ 27(71.05%)
Alcohol consumption Yes No	17 (42.50%)/ 23 (57.50%)	15 (40.00%)/ 23 (60.52%)
Sedentary lifestyle	24 (60.00%)	20 (52.63%)
CEAP classification	C1 = 18 (45.00%) C2 = 22 (55.00%)	N/A
Family history of CVD	31(77.50%)	15 (39.47%)

**Table 2:** A summary of altered cytokines found in our study, mechanisms of action, and potential implications in the event of chronic venous disease (CVD).

Cytokines/Chemokines	Status in CVD in Our Study	Mechanisms of Action	Potential Implications in CVD	References
IL-1β	Increased	Pro-inflammatory; central mediator in inflammatory cascade, promotes endothelial activation and increases vascular permeability	Enhances immune cell infiltration, contributing to endothelial dysfunction and vascular wall damage	[40,41,42,43,44,45,46,47]
TNF-α	Increased	Pro-inflammatory; works synergistically with IL-1β in inflammatory responses	Increases immune cell recruitment and endothelial activation, exacerbating vascular inflammation	[40,41,42,43,44,45,46,47]
IL-6	Increased	Induces acute inflammation proteins, directs leukocyte trafficking, promotes T-cell and B-cell responses	Linked to CVD-related changes like hemodynamic shifts, hypoxia, oxidative stress, and endothelial dysfunction	[40,48,49,50,51]
IL-17A	Increased	Drives inflammation by inducing other pro-inflammatory cytokines, recruits neutrophils and macrophages	Th17 pathway activation, associated with chronic inflammation and tissue damage in vascular walls	[52,53,54,55,56,57]
IL-23	Increased	Supports Th17 cell survival and differentiation, amplifying inflammatory responses	Th17 pathway activation, associated with chronic inflammation and tissue damage in vascular walls	[52,53,54,55,56,57]
IL-8 (CXCL8)	Increased	Neutrophil migration and recruitment, inflammatory mediator	Increases leukocyte recruitment, contributing to venous inflammation and vascular remodeling	[58,59,60,61,62,63,64]
Fractalkine (CX3CL1)	Increased	Facilitates NK, monocyte, and T-cell trafficking within vascular tissues	Favors monocyte accumulation, which may drive vascular inflammation and remodeling	[58,59,60,61,62,63,64]
ITAC (CXCL11)	Increased	Attracts activated T cells and macrophages, involved in Th1 response	Promotes immune cell recruitment to vascular lesions, potentially accelerating CVD progression	[58,59,60,61,62,63,64]
IL-5	Increased	Supports B-cell differentiation and eosinophil activation	Promotes eosinophilic inflammation and tissue remodeling within venous structures	[65,66,67,68,69,70,71,72]
GM-CSF	Increased	Eosinophil activation	Promotes eosinophilic inflammation and tissue remodeling within venous structures	[65,66,67,68,69,70,71,72]
IL-2	Increased	Expands T-cell populations, maintains immune regulation, influences Treg cells	May contribute to T cell-driven vascular inflammation and endothelial dysfunction	[19,73,74,75,76,77,78,79]
IL-7	Increased	Promotes T- and B-cell development and NK cell homeostasis	Involved in macrophage recruitment, potentially intensifies inflammation in the venous wall	[19,73,74,75,76,77,78,79]
IFN-γ	Increased	Activates Th1 response	Drives vascular inflammation and endothelial impairment, and possibly decreases nitric oxide, contributing to CVD progression	[15,80,81,82,83,84,85,86]
IL-12	Increased	Promotes Th1 cell differentiation and IFN-γ production	Supports chronic Th1 responses and endothelial dysfunction	[15,80,81,82,83,84,85,86]
IL-13	Decreased	Anti-inflammatory; promotes tissue repair and fibrosis	Diminished repair, possibly contributes to vascular wall degradation	[47,87,88,89]

## Data Availability

The data used to support the findings of the present study are available from the corresponding author upon request.

## Supplementary Materials

The following supporting information can be downloaded at: https://www.mdpi.com/article/10.3390/biomedicines13010150/s1, Table S1: Descriptive summary of multiplex analysis.
