# Supplementary material for: Evidence of Inflammatory Network Disruption in Chronic Venous Disease: An Analysis of Circulating Cytokines and Chemokines
Source: Biomedicines. 2025 Jan 9;13(1):150. doi: 10.3390/biomedicines13010150 (PMC11763091; doi:10.3390/biomedicines13010150)
Supplement: Supplementary file 1 [file biomedicines-13-00150-s001.zip › biomedicines-3383882-supplementary.pdf]

biomedicines MDPI

Table S1. Descriptive summary of multiplex analysis.

Protein marker	Mean ± sd (HC± sd CVD± sd)	Median	IQR (Q1-Q3)	p value (U Mann Whitney)
**Cytokines**				
Pro-inflammatory				
TNF-α	20.3 ± 21.59<br>45.98 ± 34.46	16.83<br>38.76	[8.32, 23.1]<br>[20.7, 59.4]	2.196e-05<br>***
IFN-γ	20.36 ± 15.91<br>37.48 ± 21.59	25.12<br>31.51	[7.90, 29.5]<br>[23.4, 46.9]	0.001051<br>**
IL1β	2.06 ± 1.24<br>2.19 ± 1.48	1.56<br>1.98	[1.01, 2.72]<br>[0.93, 2.66]	0.02197<br>*
IL12	2.55 ± 2.19<br>5.74 ± 3.08	2.62<br>4.69	[0.37, 3.77]<br>[3.94, 6.42]	2.88e-07<br>***
IL17A	9.76 ± 9.12<br>12.72 ± 7.57	8.33<br>11.54	[1.16, 12.4]<br>[8.72, 14.8]	0.02197<br>*
IL23	380.03 ± 374.77<br>553.37 ± 441.88	292.88<br>473.39	[48.6, 571]<br>[224., 652.]	0.03612<br>*
IL6	1.98 ± 2.14<br>45.21 ± 92.92	1.05<br>3.7	[0.36, 2.94]<br>[1.48, 17.2]	8.659e-05<br>***
Immunomodulatory and growth factors				
IL2	3.07 ± 2.08<br>8.46 ± 11.33	2.59<br>5.9	[0.9, 4.46]<br>[4.10, 7.60]	4.622e-07<br>***
IL21	7.91 ± 6.09<br>8.78 ± 5.9	6.99<br>7.3	[3.52, 10.8]<br>[4.67, 12.1]	0.4295
IL5	6.15 ± 11.49<br>12.28 ± 18.05	3.94<br>6.28	[0.695, 6.92]<br>[4.41, 10.9]	0.007804<br>**
IL7	10.94 ± 8.21<br>18.02 ± 26.01	9.99<br>12.54	[4.95, 14.6]<br>[10.1, 18.4]	0.02748<br>*
GMCSF	28.13 ± 24.82<br>42.72 ± 20.27	22.93<br>43.11	[8.58, 36.4]<br>[34.3, 49.5]	0.001099<br>**
Anti-inflammatories				
IL4	92.25 ± 229.72<br>91.49 ± 237.81	19.18<br>25.66	[5.40, 33.8]<br>[10.4, 49.0]	0.2523
IL-10	13.09 ± 12.41<br>16.07 ± 14.52	9.86<br>9.87	[3.55, 19.1]<br>[4.62, 26.4]	0.3675
IL-13	82.74 ± 189.54<br>13.48 ± 44.3	7.55<br>2.95	[2.51, 28.9]<br>[0.53, 12.3]	0.01481<br>*
**Chemokines**				
ITAC	42.29 ± 25.56<br>117.54 ± 145	34.77<br>69.28	[22.9, 53.4]<br>[38.3, 115.]	0.0004668<br>***
Fractalkine (CX3CL1)	128.53 ± 78.05<br>225.21 ± 76.89	128.85<br>212.75	[43.3, 184.]<br>[180., 251.]	5.188e-06<br>***
MIP1α	201.34 ± 478.82<br>184.68 ± 352.62	9.4<br>48.7	[5.08, 90.0]<br>[29.4, 91.1]	0.06962
MIP1β	5.29 ± 4.98<br>5.34 ± 3.71	3.43<br>4.58	[2.65, 6.86]<br>[3.01, 7.33]	0.3927
MIP3α	35.54 ± 20.48	29.7	[26.2, 34.0]	0.4473

	42.08 ± 43.28	30.59	[27.1, 41.5]	
IL8	7.7 ± 2.97	6.76	[5.62, 9.69]	0.0008403
	11.05 ± 5.07	10.66	[6.42, 14.8]	***

N (HC) = 38, N (CVD) = 40. Numerical values express concentration of each protein in pg/mL. Mean values for each protein marker (proinflammatory cytokines, anti-inflammatory cytokines and chemokines) are expressed with their standard deviations (sd). Median values and interquartile range (IQR) expressed with quartile 1 (Q1) and quartile 3 (Q3) are also described. Results from the U Mann Whitney statistical test are addressed to reveal if the differences among healthy control (HC) and Chronic Venous Disease (groups) are significant. p value: ***<0.001, **<0.01, *<0.05.
